# Identifiability and convergence behavior for Markov chain Monte Carlo using multivariate probit models

**DOI:** 10.1080/03610926.2024.2425738

**Published:** 2024-11-09

**Authors:** Xiao Zhang

**Affiliations:** Department of Mathematical Sciences, Michigan Technological University, Houghton, MI, USA

**Keywords:** Identifiability, MCMC, multivariate probit model, parameter expansion

## Abstract

Multivariate probit models have been popularly utilized to analysis multivariate ordinal data. However, the identifiable multivariate probit models entail the covariance matrix for the underlying multivariate normal variables to be a correlation matrix, which brings a rigorous task to conduct efficient statistical analysis. Parameter expansion to make the identifiable model to be non-identifiable has been inevitably explored. However, the effect of the expanded parameters on the convergence of Markov chain Monte Carlo (MCMC) is seldomly investigated; in addition, the comparison of MCMC developed based on the identifiable model and that based on the non-identifiable model is hardly ever explored, especially for data with large sample sizes. In this paper, we conduct a thorough investigation to illustrate the effect of the expanded parameters on the convergence of MCMC and compare the behavior of MCMC between the identifiable and non-identifiable models. Our investigation provides a practical guide regarding the construction of non-identifiable models and development of corresponding MCMC sampling methods. We conduct our investigation using simulation studies and present an application using data from the Russia Longitudinal Monitoring Survey-Higher School of Economics (RLMS-HSE) study.

## 1. Introduction

Probit models assume that binary/ordered categorical data are discretized versions of the underlying normal distributed variables (Pearson 1900). Ashford and Sowden (1970) extended probit models for univariate binary/ordinal data to multivariate probit models for multivariate binary/ordinal data. Since then, multivariate probit models are popularly utilized to analyze longitudinal/multivariate binary/ordinal data in statistical field, especially in Bayesian statistics due to inexplicit likelihood functions. However, the identifiable multivariate probit models require the covariance matrix for each underlying multivariate normal variable to be a correlation matrix (Albert and Chib 1993), and this model identification issue hinders to develop efficient MCMC algorithms. Various Metropolis-Hastings (MH) algorithms have been developed, such as Chib and Greenberg (1998), Liu and Daniels (2006), and Zhang, Boscardin, and Belin (2006), which produce MCMC with slow convergence or necessitate limited prior distributions for the correlation matrix.

Liu and Wu (1999) proposed parameter expansion to construct non-identifiable models and proved that under mild conditions the data augmentation based on the expanded/non-identifiable models converges no slower than the original data augmentation. The parameter expansion has also been explored by Liu, Rubin, and Wu (1998), Meng and van Dyk (1999), and Lewandowski, Liu, and Wiel (2010) for the expectation-maximization algorithm, while Nobile (1998) and Imai and van Dyk (2005) examined this issue for univariate categorical data using multinomial probit models. Lawrence et al. (2008) and Zhang (2022) developed the parameter-expanded data augmentation to analyze the multivariate ordinal data using multivariate probit model, while Zhang (2020) did for multivariate binary data. Other related work can be referred to Edwards and Allenby (2003), MacEachern (2007), and Van Dyk (2010).

Eberly and Carlin (2000) investigated the identifiability and convergence of MCMC for spatial models and Shariati, Korsgaard, and Sorensen (2009) did the investigation using the reaction norm model. However, the identifiability and convergence issue of the expanded multivariate probit models have been rarely investigated. In this paper, we conduct a thorough investigation to illustrate the effect of the expanded parameters on the convergence of MCMC components and compare the performance of MCMC samplings between the identifiable and non-identifiable multivariate probit models. The reminder of this paper is organized as follows. Section 2 presents the identifiable multivariate probit model for multivariate ordinal data and the parameter-extended Metropolis-Hastings (PX-MH) algorithm (Zhang, Boscardin, and Belin 2006). In Section 3, we present the non-identifiable multivariate probit model for multivariate ordinal data and two MCMC sampling algorithms (Zhang 2022). Simulation studies are conducted in Section 4 to investigate the effect of expanded parameters in the convergence of MCMC and compare the MCMC algorithms based on the identifiable and non-identifiable probit models. For illustration, we present an application using the data from the RLMS-HSE study in Section 5. Discussion and conclusions are offered in Section 6.

## 2. Multivariate probit model for multivariate ordinal data and the PX-MH algorithm

Albert and Chib (1993) first introduced the univariate probit model, which assumes that there is an underlying univariate normal variable corresponding to an ordinal variable. Specifically, suppose there are n individuals and each individual has an ordinal outcome $Y_{i}$ with J ordinal categories and a p×1 covariate vector $X_{i}$ for i=1,⋯,n. The probit model assumes that there is a latent variable $Z_{i}$ underlying $Y_{i}$, following a normal distribution with mean $X_{i}\beta$ and variance being $\sigma^{2}$, denoted by $N\left(X_{i}^{T}\beta ,\sigma^{2}\right)$, where β is the p×1 regression parameter vector. The model further assumes that

$$Y_{i}=l\Leftrightarrow \gamma_{l-1}<Z_{i}\leq \gamma_{l}\;\text{for}\;l=1,\ldots ,J,$$

i.e., $P\left(Y_{j}\leq l\right)=\text{Φ}\left(\frac{\gamma_{l}-X_{i}^{T}\beta }{\sigma }\right)$, where Φ(⋅) is the standard normal distribution function and $\gamma =\left(\gamma_{0},\gamma_{1},\ldots \gamma_{J}\right)$ being the unknown cut-points. It is noticeable that the model is non-identifiable due to β, σ and $\gamma_{0},\gamma_{1},\ldots \gamma_{J}$ being unknown parameters. It is customary to define that $\sigma^{2}=1$ and $\gamma_{0}=-\infty$, $\gamma_{1}=0$, and $\gamma_{J}=\infty$ for the model identification purpose.

Chib and Greenberg (1998) extended the univariate probit model to multivariate probit model. As a matter of course the multivariate probit model assumes that there is an underlying multivariate normal variable corresponding to a multivariate ordinal variable. Assume for each individual i, $Y_{i}={\left(Y_{i1},\ldots ,Y_{ik}\right)}^{T}$ is the ordinal outcome composed of k ordinal measures $Y_{i1},\cdots ,Y_{ik}$ with each $Y_{ij}$ has $J_{j}$ ordinal categories for i=1,⋯,n and j=1,…,k; $X_{i}={\left(X_{i1},\cdots ,X_{ik}\right)}^{T}$ is the covariate matrix for individual i. Then, the univariate probit model is still assumed for each $Y_{ij}$ by

(1) $$Y_{ij}=l\Leftrightarrow \gamma_{j,l-1}<Z_{ij}\leq \gamma_{j,l}\;\text{for}\;l=1,\ldots ,J_{j},$$

where $Z_{ij}$ is the underlying latent variable following $N\left(X_{ij}^{T}\beta ,1\right)$, and $\gamma_{j}=\left(\gamma_{j,0},\gamma_{j,1},\ldots \gamma_{j,J_{j}}\right)$ is the unknown cut-points with $\gamma_{j,0}=-\infty$, $\gamma_{j,1}=0$, and $\gamma_{J_{j}}=\infty$. With each component $Y_{ij}$, j=1,…,k, collected from individual i, they are correlated as expected. Therefore, the latent variable $Z_{i}={\left(Z_{i1},\ldots ,Z_{ik}\right)}^{T}$ underlying $Y_{i}={\left(Y_{i1},\ldots ,Y_{ik}\right)}^{T}$ is correlated. With the variance for each latent component $Z_{ij}$ being fixed at 1 due to the model identification issue, $Z_{i}={\left(Z_{i1},\ldots ,Z_{ik}\right)}^{T}$ is assumed to follow a multivariate normal distribution with the mean vector being $X_{i}\beta$ and the covariance matrix R, which in fact is a correlation matrix, i.e., $Z_{i}∼N_{k}\left(X_{i}\beta ,R\right)$. The covariance matrix R actually being a correlation matrix is discussed and elaborated in detail by Albert and Chib (1993) and Chib and Greenberg (1998). The correlation matrix R is called the polychoric correlation matrix of $Y_{i}$ (Drasgow 2004).

The unknown quantities in the model contain the regression parameter vector β, the cut-points $\gamma =\left(\gamma_{1},\cdots ,\gamma_{k}\right)$, the correlation matrix R and the latent multivariate normal variables $Z=\left(Z_{1},\cdots ,Z_{n}\right)$. We assume the priors for β, γ and R are independent, i.e., P(β,γ,R)=P(β)×P(γ)×P(R). Then the posterior joint density of β, γ, R, Z given the observed multivariate ordinal outcome $Y=\left(Y_{1},\cdots ,Y_{n}\right)$ can be derived in the following:

$$\begin{array}{l} P(\beta ,\gamma ,R,Z|Y)\propto P(\beta )\times P(\gamma )\times P(R)\times P(Z|\beta ,\gamma ,R,Y)\\ \;\propto P(\beta )\times P(\gamma )\times P(R)\times \prod_{i=1}^{n}\left[I_{i}\times \phi \left(Z_{i};X_{i}\beta ,R\right)\right] \end{array}$$

where ϕ(⋅) is the standard normal density function, and $I_{i}=\prod_{j=1}^{k}I_{ij}$, where $I_{ij}=\sum_{t=1}^{J_{j}}1_{\left(Y_{ij}=t\right)}1_{\left(\gamma_{j}(t-1)<Z_{ij\leq }\gamma_{jt}\right)}$), indicating compatibility of the latent variable $Z_{ij}$ with the ordinal variable $Y_{ij}$ defined in (1).

To implement the MCMC sampling, each full conditional distribution is listed as follows:

- $\beta |\gamma ,R,Z,Y∼N_{k}\left(\hat{\beta },V_{\beta }\right)$, where $V_{\beta }={\left(\sum_{i=1}^{n}X_{i}^{T}R^{-1}X_{i}+C^{-1}\right)}^{-1}$ and $\hat{\beta }=V_{\beta }\left(\sum_{i=1}^{n}X_{i}^{T}R^{-1}Z_{i}+C^{-1}b\right)$ assuming the prior of β follows $N_{p}(b,\text{C})$ with the mean vector equal to b and the covariance matrix equal to C.
- $Z_{ij}|\beta ,\gamma ,R,Y,Z_{ik},k\neq j$ has interval truncated normal distribution constrained to lie between the two cut-points $\gamma_{j,l-1}$ and $\gamma_{j,l}$, assuming $Y_{ij}=l$, based on Albert and Chib (1993).
- $\gamma_{j,l}|\beta ,R,Z,Y,\gamma_{j,k},k\neq l$, from a uniform distribution, $U\left(\gamma_{j,l}|\max\right.\left\{\max\left[Z_{ij}:Y_{ij}=l\right],\gamma_{j,l-1}\right\},\left.\min\left\{\min\left[Z_{ij}:Y_{ij}=l+1\right],\gamma_{j,l+1}\right\}\right)$, based on Albert and Chib (1993).
- The full conditional density function of R can be derived as $P(R|\beta ,\gamma ,Z,Y)\propto P(R)\times \prod_{i=1}^{n}\phi \left(Z_{i};X_{i}\beta ,R\right)$. As evident, it is not possible to directly sample R, which is a correlation matrix instead of a covariance matrix, and it is problematic to choose a prior distribution for R as well. Various MH algorithms have been developed to sample R (Chib and Greenberg 1998; Liu and Daniels 2006). Here we present the MCMC sampling algorithm proposed by Zhang, Boscardin, and Belin (2006).

Realizing the difficulty to specify a prior for R, a joint prior of R and an artificial diagonal matrix D with diagonal elements serving as variance parameters is derived by assuming $\text{Σ}=D^{1/2}RD^{1/2}$ follows a Wishart distribution with m degrees of freedom and scale matrix V, i.e., $\text{Σ}∼Wishart_{k}(V,m)$; then $P(R,D)=P(\text{Σ})\times \left|J_{\left(\text{Σ}\right)}(R,D)\right|$ with Jacobian matrix $\left|J_{\left(\text{Σ}\right)}(R,D)\right|=|D{|}^{\frac{k-1}{2}}$.

Including artificial diagonal matrix D, the posterior joint density of β, γ, R, D, Z given Y is

$$\begin{array}{l} P(\beta ,\gamma ,R,D,Z|Y)\propto P(\beta )\times P(\gamma )\times P(R,D)\times P(Z|\beta ,\gamma ,R,Y)\\ \;\propto P(\beta )\times P(\gamma )\times P(R,D)\times \prod_{i=1}^{n}\left[I_{i}\times \phi \left(Z_{i};X_{i}\beta ,R\right)\right]. \end{array}$$

As can be seen, the posterior inference for β, γ, and Z does not change and D only facilitates to specify a prior for R. Therefore, the model remains identifiable. The following MH algorithm is proposed to sample R and D :

Set the initial value of $\left(R^{\left(0\right)},D^{\left(0\right)}\right)$ through setting ${\text{Σ}}^{\left(0\right)}=D^{(0)\frac{1}{2}}R^{\left(0\right)}D^{(0)\frac{1}{2}}$ to an initial covariance matrix. Then, at iteration t

1. Generate $D^{*}$ by generating ${\text{Σ}}^{*}=D^{*\frac{1}{2}}R^{*}D^{*\frac{1}{2}}$ from $\text{Wishar}{\text{t}}_{k}\left({\text{Σ}}^{\left(t\right)}/m_{p},m_{p}\right)$.
2. Take
$$\left(R^{\left(t+1\right)},D^{\left(t+1\right)}\right)=\left\{\begin{array}{l} \left(R^{*},D^{*}\right)\;\text{with probability}\;\alpha \\ \left(R^{\left(t\right)},D^{\left(t\right)}\right)\;\text{otherwise}\;, \end{array}\right.$$
  where $\alpha =\min\left\{\frac{P\left(R^{*},D^{*}|\beta ,\gamma ,Z,Y\right)}{P\left(R^{\left(t\right)},D^{\left(t\right)}|\beta ,\gamma ,Z,Y\right)}\frac{f\left({\text{Σ}}^{\left(t\right)}|{\text{Σ}}^{*}\right)}{f\left({\text{Σ}}^{*}|{\text{Σ}}^{\left(t\right)}\right)},1\right\}$, and the proposal density $f\left({\text{Σ}}^{*}|{\text{Σ}}^{\left(t\right)}\right)$ is equal to
$$J_{{\text{Σ}}^{*}\to R^{*},D^{*}}\times \text{Wishar}{\text{t}}_{k}\left({\text{Σ}}^{\left(t\right)}/m_{p},m_{p}\right).$$

The artificial diagonal matrix D aids in the prior specification for R and the MH algorithm to sample R without altering the model’s identifiability and this MCMC sampling algorithm is termed as the PX-MH algorithm.

We also considered to transform the ordered cut-points to make them unordered used by Albert and Chib (2001) and to tailor the parameters of the proposal distribution suggested by Chib and Greenberg (1995) to improve the convergence of the MCMC algorithm and did not find significant difference. In the following discussion we use the PX-MH algorithm to compare with those developed based on the non-identifiable multivariate probit model.

## 3. The non-identifiable multivariate probit models and the MCMC sampling algorithms

The identifiable multivariate probit model described in Section 2 assumes that *$Z_{i}$*, the underlying multivariate normal variable, follows $N_{k}\left(X_{i}\beta ,R\right)$, i.e., $Z_{i}∼N_{k}\left(X_{i}\beta ,R\right)$. i=1,…n. To construct the non-identification in the model, Zhang (2022) assumes $Z_{i}∼N_{k}\left(D^{-1/2}X_{i}\beta ,R\right)$ instead of $Z_{i}∼N_{k}\left(X_{i}\beta ,R\right)$, where D is the diagonal matrix with the diagonal elements $d=\left(\sigma_{11},\sigma_{22},\cdots ,\sigma_{kk}\right)$ and $\sigma_{jj}>0$ for j=1,…k. As noted, by including D in this way, the identifiable model becomes non-identifiable. We then augment $Z_{i}$ to be $W_{i}$ by $W_{i}=D^{1/2}Z_{i}$ with $W_{i}∼N_{k}\left(X_{i}\beta ,D^{1/2}RD^{1/2}\right)$. It is evident that $D^{1/2}RD^{1/2}$ is a covariance matrix without restrictions on the diagonal elements, and we denote $\text{Σ}=D^{\frac{1}{2}}RD^{\frac{1}{2}}$. Then the multivariate probit model can be defined based on $W_{i}$ with $W_{i}∼N_{k}\left(X_{i}\beta ,\text{Σ}\right)$, i=1,⋯,n, as follows:

(2) $$Y_{ij}=l\Leftrightarrow \zeta_{j,l-1}<W_{ij}\leq \zeta_{j,l},$$

with $\zeta_{j}=\left(\zeta_{j,0},\zeta_{j,1},\ldots \zeta_{j,J_{j}}\right)$ being the unknown cut-points with $\zeta_{j,0}=-\infty$, $\zeta_{j,1}=0$, and $\zeta_{J_{j}}=\infty$, for j=1,…,k. We assume an independent prior distribution for β, ζ, and Σ, i.e., P(β,ζ,Σ)=P(β)×P(ζ)×P(Σ). Then, the joint posterior density of β, ζ, Σ, and W given Y can be derived in the following:

P(β,ζ,Σ,W∣Y)

$$\propto \left(\prod_{i=1}^{n}I_{i}\right)\times P(\beta )\times P(\zeta )\times P(\text{Σ})\times |\text{Σ}{|}^{-\frac{n}{2}}$$

$$\;\times \exp\left[-\frac{1}{2}\sum_{i=1}^{n}{\left(W_{i}-X_{i}\beta \right)}^{T}{\text{Σ}}^{-1}\left(W_{i}-X_{i}\beta \right)\right],$$

where $I_{i}=\prod_{j=1}^{k}I_{ij}$ and $I_{ij}=\sum_{t=1}^{J_{j}}1_{\left(Y_{ij}=t\right)}1_{\left(\zeta_{j}(t-1)<w_{ij\leq }\zeta_{jt}\right.}$, indicating compatibility of the latent variable $W_{ij}$ with the ordinal variable $Y_{ij}$ defined in (2).

We set the prior of β as $N_{p}(b,\text{C})$, non-informative prior for each component of ζ, and an inverse-Wishart distribution for the prior of Σ, i.e., $P(\text{Σ})=\text{InvWis}{\text{h}}_{k}(V,m)$, which is a conjugate prior for Σ. Then the MCMC sapling algorithm can be derived as follows:

- $\beta |\text{Σ},W,Y∼N_{k}\left(\hat{\beta },V_{\beta }\right)$, where $\hat{\beta }$ and $V_{\beta }$ are defined in the sampling β in Section 2 by replacing Z with W and R with Σ.
- $W_{ij}|\beta ,\zeta ,\text{Σ},Y,W_{ik}$, k≠j, from a truncated normal distribution constrained to lie between the two cut-points $\zeta_{j,l-1}$ and $\zeta_{j,l}$, assuming $Y_{ij}=l$, based on Albert and Chib (1993).
- $\zeta_{j,l}|\beta ,\text{Σ},W,Y$, $\zeta_{j,k}$, k≠l, from a uniform distribution,
- $U\left(\zeta_{j,l}|\max\left\{\max\left[W_{ij}:Y_{ij}=l\right],\zeta_{j,l-1}\right\}\right.$, $\left.\min\left\{\min\left[W_{ij}:Y_{ij}=l+1\right],\zeta_{j,l+1}\right\}\right)$, based on Albert and Chib (1993).
- $\text{Σ}|\beta ,\zeta ,W,Y∼\;{\text{Inverse-Wishart}}_{k}\left(\sum_{i=1}^{n}\left(W_{i}-X_{i}\beta \right){\left(W_{i}-X_{i}\beta \right)}^{T}+V,n+m+\right.\left.k+1\right)$.

As has been noted, the above sampling of Σ is a Gibbs sampling instead of an MH sampling for the correlation matrix R for the identifiable model. Hence, this sampling algorithm is termed as the parameter-expanded Gibbs sampling (PX-GS) algorithm (Zhang 2022). As a consequence, the regression parameter vector β and cut-points in ζ are not identifiable.

With the diagonal elements of D as the redundant parameters, marginalizing D is considered to improve the convergence and mixing of the PX-GS algorithm (Liu 1994, Van Dyk 2010). We present the following sampling steps:

- W, D∣β,ζ,R,Y by sampling D∣β,ζ,R,Y and then W∣β,ζ,R,D,Y;
- β,ζ,R,D∣W,Y by sampling β∣R,D,W,Y, followed by R,D∣β,W,Y, and then ζ∣β,R,D,W,Y.

This algorithm is termed as the parameter-expanded Gibbs sampling with marginalization (PX-GSM) algorithm (Zhang 2022). Notice that $P(D|\beta ,\zeta ,R,Y)=P(D|R)\propto P(R,D)=P(\text{Σ})\times \left|J_{\left(\text{Σ}\right)}(R,D)\right|$ with $\text{Σ}∼\text{Wishar}{\text{t}}_{k}(V,m)$. If V is a diagonal, then the diagonal elements of D, $d=\left(\sigma_{11},\sigma_{22},\cdots ,\sigma_{kk}\right)$, are independent, and $\sigma_{jj}$ follows an inverse-Gamma a $\left(\alpha =\frac{m}{2},\beta =\frac{2}{V_{jj}\tilde{r_{jj}}}\right)$ with $V_{jj}$ being the jth element of V and $\tilde{r_{jj}}$ being the jth diagonal element of inverse of R for j=1,⋯,k. However, if V is not a diagonal matrix, sampling D necessitates an MH algorithm. Based on Zhang (2020, 2022), with sample size being 500, the posterior inference is robust to the prior specification. Therefore, we set the prior of Σ, $P(\text{Σ})={\;\text{Inverse-Wishart}\;}_{k}(V,m)$ with V being a diagonal matrix, and non-informative priors for β, γ, and ζ, in the following discussion for sample sizes being 500 and 2000 as well.

## 4. Simulation studies

There are three purposes to conduct simulation studies for the investigation of the PX-MH algorithm based on the identifiable model (Section 2) and the PX-GS and PX-GSM algorithms based on the non-identifiable model (Section 3). First, we want to examine the estimated quantities of the regression parameter vector, the cut-points, and the correlation matrix from these three algorithms; secondly, we want to investigate their convergence behaviors by comparing the PX-MH algorithm based on the identifiable model *vs.* the PX-GS and PX-GSM algorithms based on non-identifiable models; lastly, we investigate the convergence behavior of redundant parameters and examine the PX-GS and PX-GSM algorithms for the effect of marginalization.

We generate 5-dimensional ordinal data with 4 categories in each dimension. Based on the model definition in Section 2, there are 3 cut-points for 4 categories with the first cut-point fixed at 0; then we set the other two cut-points for each dimension with values listed in Table 1. We assume the covariate matrix to be the identity matrix for each individual and fixed for all the generations; and the true values for each regression parameters can be referred to Table 1. The identifiable values for $\beta_{j}$, are calculated as $\frac{\hat{\beta_{j}}}{\sqrt{\sigma_{jj}}}$ and the identifiable cut-points as $\frac{\hat{\zeta_{j1}}}{\sqrt{\sigma_{jj}}}$ and $\frac{\hat{\zeta_{j2}}}{\sqrt{\sigma_{jj}}}$ for j=1,⋯,5. The correlation matrix $R={\left(r_{ij}\right)}_{5\times 5}$ is assumed to be autoregressive AR1(0.5) and all the redundant variance parameters $\left(\sigma_{11},\ldots ,\sigma_{55}\right)$ are set at 1.

We assume non-informative priors for regression parameter vector β and the cut-points γ or ζ. For the PX-MH algorithm, we assume the prior of R and D derived based on $\text{Wishar}{\text{t}}_{k}(I,m)$ with m=10; for PX-GS and PX-GSM algorithms, we assume the prior for Σ being Inverse-$\text{Wishar}{\text{t}}_{k}(I,m)$ with m=20. Here the I indicates the identity matrix and the values for m are chosen for weak prior specification.

We investigate sample sizes being 500 and 2000 and conduct each simulation study based on 500 generated datasets. We assess the convergence behaviors using CODA (Plummer et al. 2006) and BOA Smith 2007). We run the MCMC sampling with 20,000 iterations and 5,000 burn-in.

Tables 1 and 2 present the averaged posterior means, standard deviations, and 95% credible interval coverage probabilities based on 500 simulated data with sample sizes being 500 and 2000. It can be seen that for sample size being 500 (Table 1), all three algorithms produce similar estimated posterior quantities, except that the PX-GSM algorithm gives larger standard deviations for regression parameters $\left(\beta \right)$ and the cut-points (γ) and therefore has better 95% credible coverage probabilities (CP%) than the others. With sample size being 2,000 (Table 2), the PX-MH and PX-GSM algorithms produce similar estimated quantities except that the PX-GSM algorithm still has larger standard deviations and thus better coverage probabilities for β and γ: We notice that the PX-GS algorithm produces extremely lower coverage probabilities for $\beta_{3}$, $\beta_{4}$, $\beta_{5}$, $r_{12}$, $r_{23}$, $r_{34}$, $r_{45}$ and for all the cut-points, and gives more biased estimated values than the other two algorithms.

**Figure 1.**
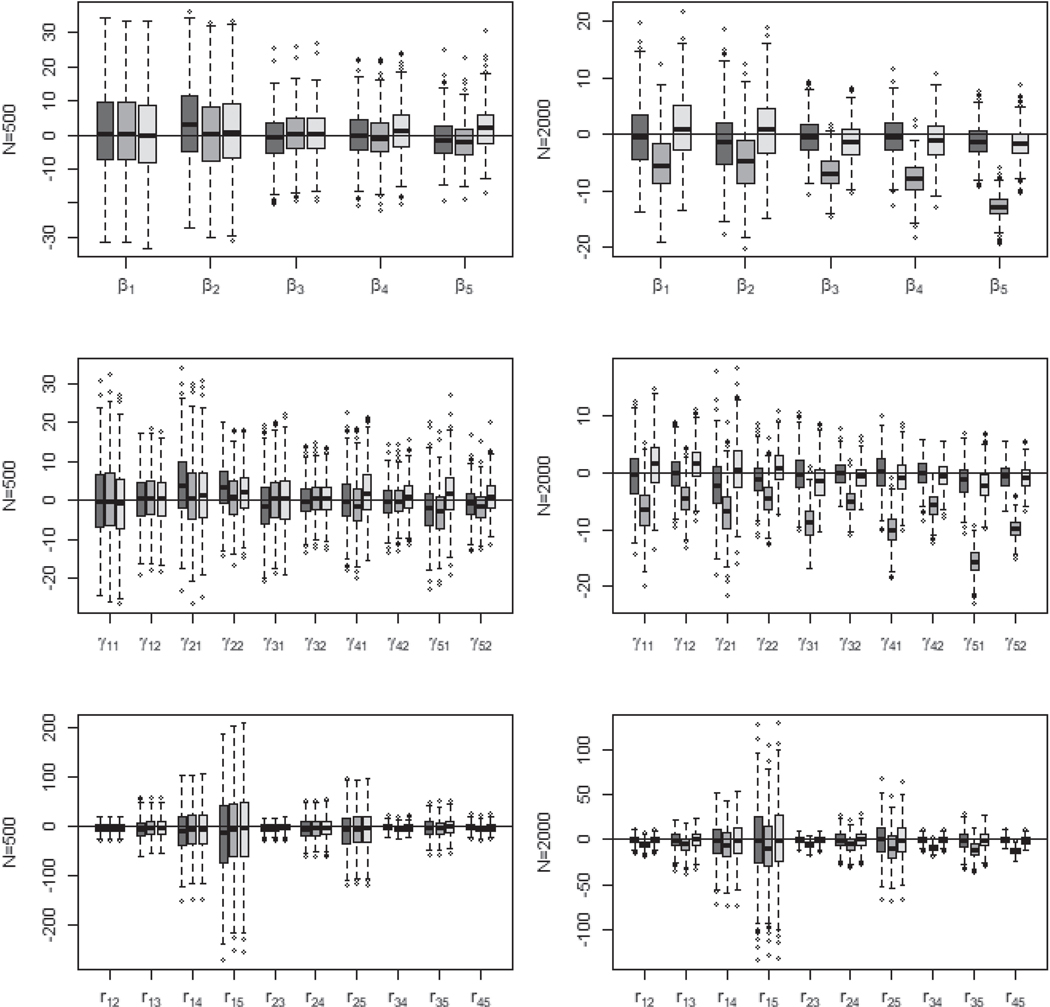
Boxplots of averaged relative bias for regression parameters, cut-points, and correlations with sample sizes being 500 (1st column) and 2000 (2nd column) based on 500 simulated datasets. The dark grey indicates the PX-MH algorithm, the medium grey indicates the PX-GS algorithm and the light grey indicates the PX-GSM algorithm.

Figure 1 provides the boxplots of averaged relative bias for regression parameters, cut-points, and correlations with sample sizes being 500 and 2000. As can be seen, the boxplots (1st column of Figure 1) with sample size being 500 are roughly similar for these three algorithms. However, it is evident for sample size being 2000 (2nd Column of Figure 1) that the PX-GS algorithm produces serious relative biases for almost all parameters, while the PX-MH and PX-GSM algorithms show similar performances.

**Figure 2.**
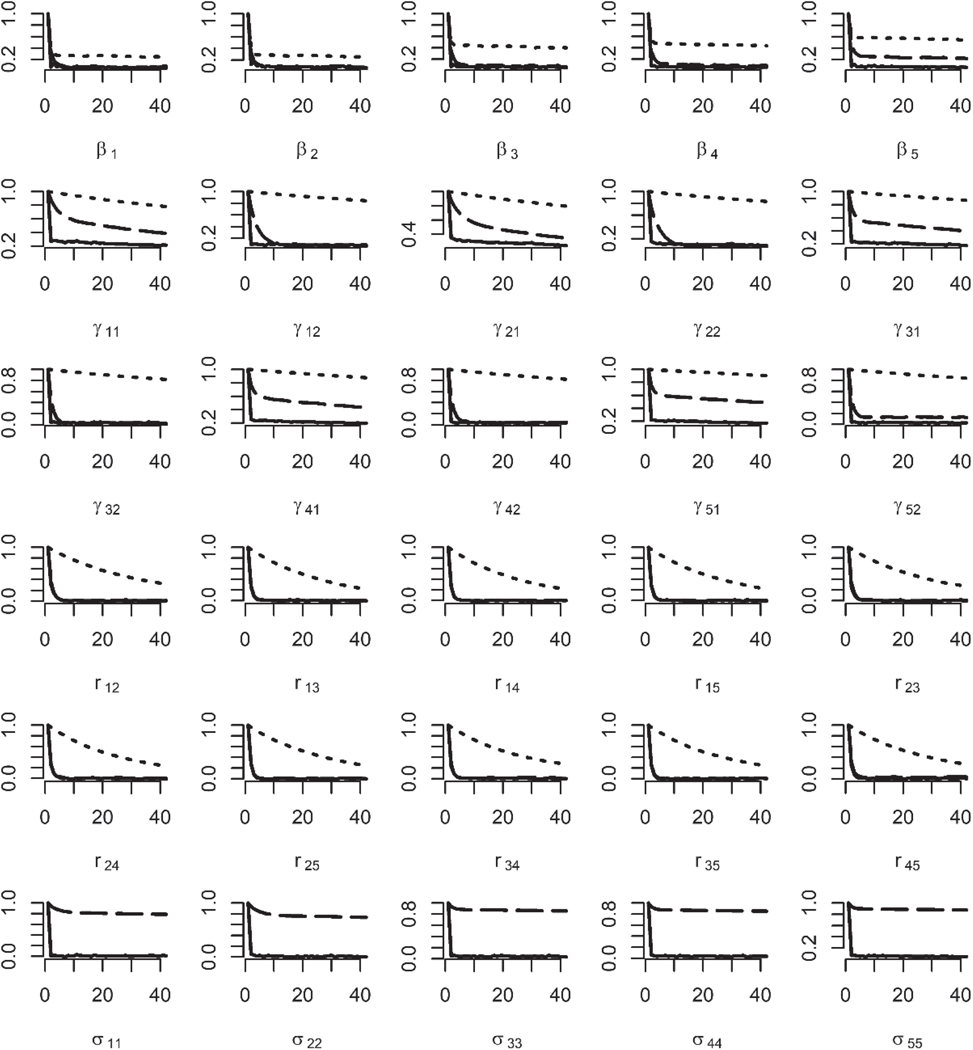
The ACF plots for regression parameters, cut-points, correlations for the PX-MH, PX-GS, and PX-GSM algorithms (first five rows), and redundant variance parameters from PX-GS and PX-GSM algorithms (last row) with sample sizes being 500 based on 500 simulated datasets. The dotted lines indicate the PX-MH algorithm, the long-dashed lines indicate the PX-GS algorithm and the solid lines indicate the PX-GSM algorithm.

Figure 2 shows the auto-correlation function (ACF) plots for sample size being 500. It is noticeable that the ACF values of the PX-GS and PX-GSM algorithms decrease faster than those of the PX-MH algorithm, suggestions that the MCMC sampling algorithms based on the non-identifiable model converge faster than that based on the identifiable model. The PX-GS and PX-GSM algorithms show similar ACF values for regression parameters, half of the cut-points $\left(\gamma_{12},\gamma_{22},\gamma_{32},\gamma_{42},\gamma_{52}\right)$ and correlations, but for the redundant variance parameters, the ACF values of the PX-GS algorithm decrease extremely slow and thus imply slow convergence of those redundant parameters.

**Figure 3.**
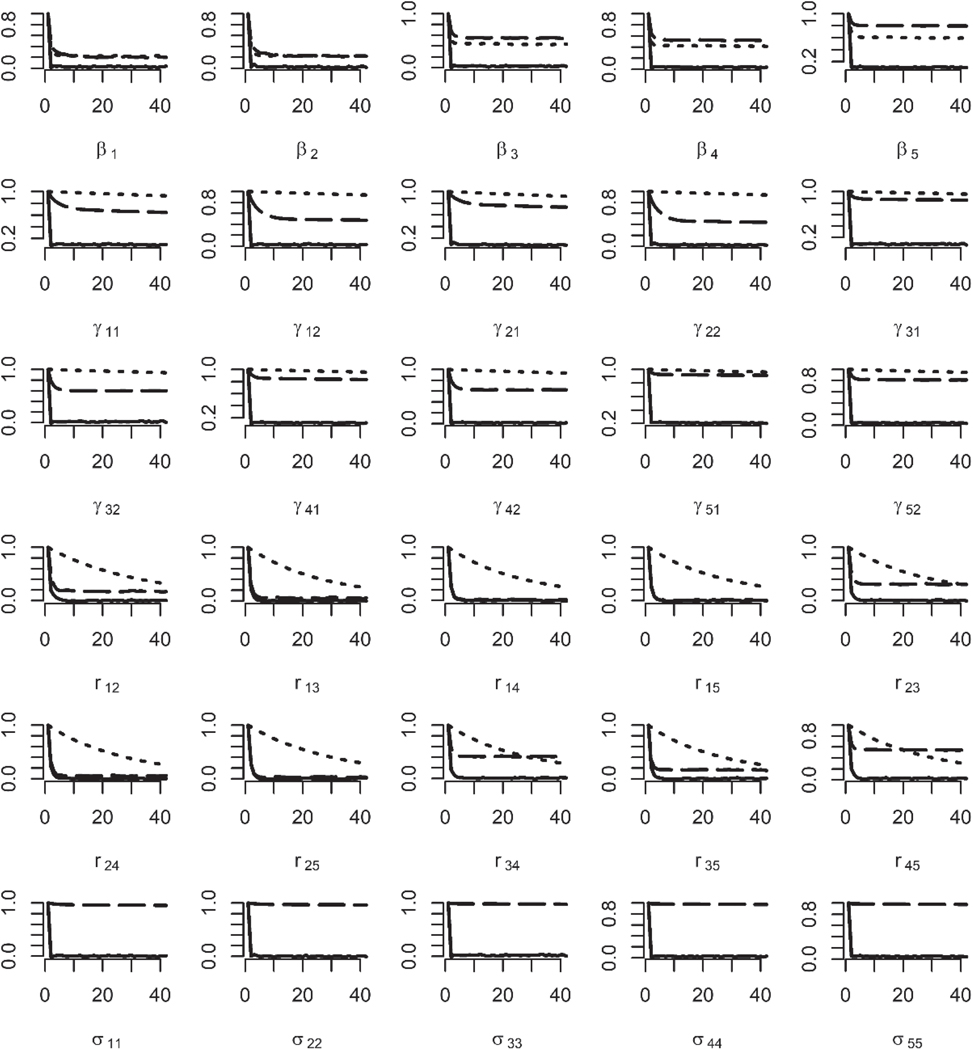
The ACF plots for regression parameters, cut-points, correlations for the PX-MH, PX-GS, and PX-GSM algorithms (first five rows), and redundant variance parameters from PX-GS and PX-GSM algorithms (last row) with sample sizes being 2000 based on 500 simulated datasets. The dotted lines indicate the PX-MH algorithm, the long-dashed lines indicate the PX-GS algorithm and the solid lines indicate the PX-GSM algorithm.

Figure 3 shows the auto-correlation function (ACF) plots for sample size being 2000. As shown, for regression parameters and cut-points, the ACF values of the PX-MH and PX-GS algorithms decrease much slower those of the PX-GSM algorithm. For correlation $r_{23}$, $r_{34}$ and $r_{45}$, the ACF values of the PX-GS algorithm decrease slower than the other two algorithms. For the redundant variance parameters, similar conclusion can be drawn as Figure 2.

**Figure 4.**
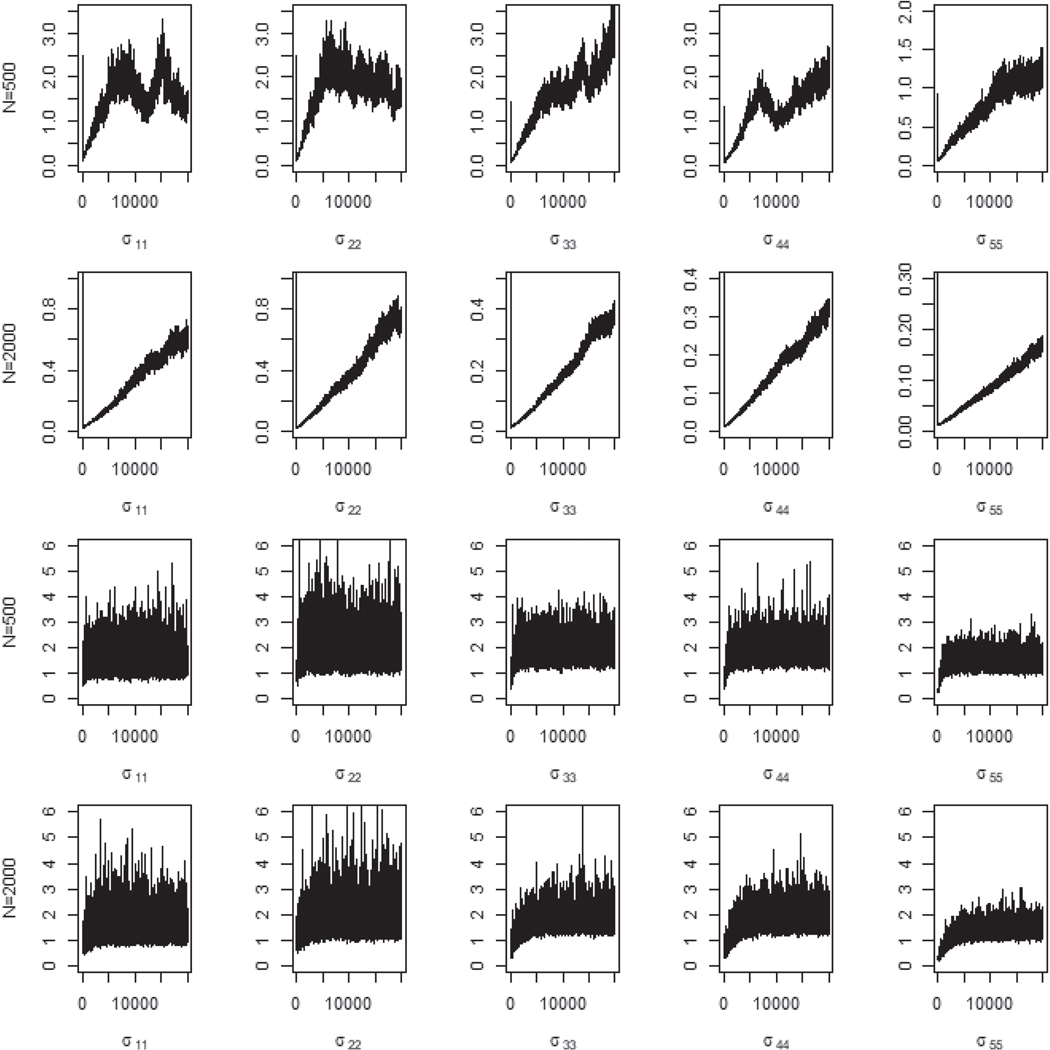
The trace plots for five redundant variance parameters with sample sizes being 500 and 2000 based on 500 simulated datasets. The first two rows are for the PX-GS algorithm and the last two rows are for the PX-GSM.

Figure 4 contains the trace plots of the redundant variance parameters for the PX-GS and PX-GSM algorithms. It is clearly seen that the PX-GSM algorithm provides good and rapid mixing for both sample sizes being 500 and 2000, yet the PX-GS algorithm exhibits slow mixing, especially for sample size being 2000.

In conclusion, the simulation studies show that for sample size being 500, the PX-GS and PX-GSM algorithms based on the non-identifiable models outperform the PX-MH algorithm based on the identifiable model in MCMC convergence, but all three provide similar estimated quantities. For sample size 2000, the PX-GS algorithm underperforms due to extremely slow convergence of the redundant variance parameters. The PX-MH and PX-GSM algorithms have similar performances in parameter estimation, yet the PX-GSM algorithm surpasses the PX-MH algorithm in MCMC convergence.

## 5. Application to the RLMS-HSE study

The RLMS-HSE study (Kozyreva, Kosolapov, and Popkin 2016) was to collect data to study the social, health, and economic situation in Russia. One of the main aspects focuses on the job satisfaction with the primary and secondary employments, which is a 5-categorical ordinal variable with 1 “absolutely satisfied,” 2 “mostly satisfied,” 3 “neutral,” 4 “not very satisfied,” and 5 “absolutely unsatisfied.” In this investigation, we choose 7-year job satisfaction data from 2010 to 2016 with 4 categories by combining category 4 “not very satisfied” and category 5 “absolutely unsatisfied” and include gender (1 being male and 0 female), education (1 having university diploma and 0 otherwise), marital status (1 being in a registered marriage and 0 otherwise), and age as the covariates. We have a total of 2439 individuals after excluding those with missing values.

We run 20,000 iterations with 5,000 burn-in for the PX-MH, PX-GS, and PX-GSM algorithms and use the same prior specifications as those in Section 4. Table 3 presents the estimated posterior means with standard deviations and the 95% credible interval for the regression parameters. As shown, the PX-MH algorithm tends to have the largest absolute estimated means while the PX-GS algorithm tends to have the smallest absolute values; the PX-GSM algorithm gives the largest standard deviations while the PX-GS algorithm gives the smallest. For all three algorithms, the 95% credible intervals for gender include 0, suggesting that the job satisfaction score may not significantly relate to gender; while those for education, marital status, and age exclude 0, suggesting individuals with higher education, registered marriage, and being older tend to have significantly higher job satisfaction scores.

**Figure 5.**
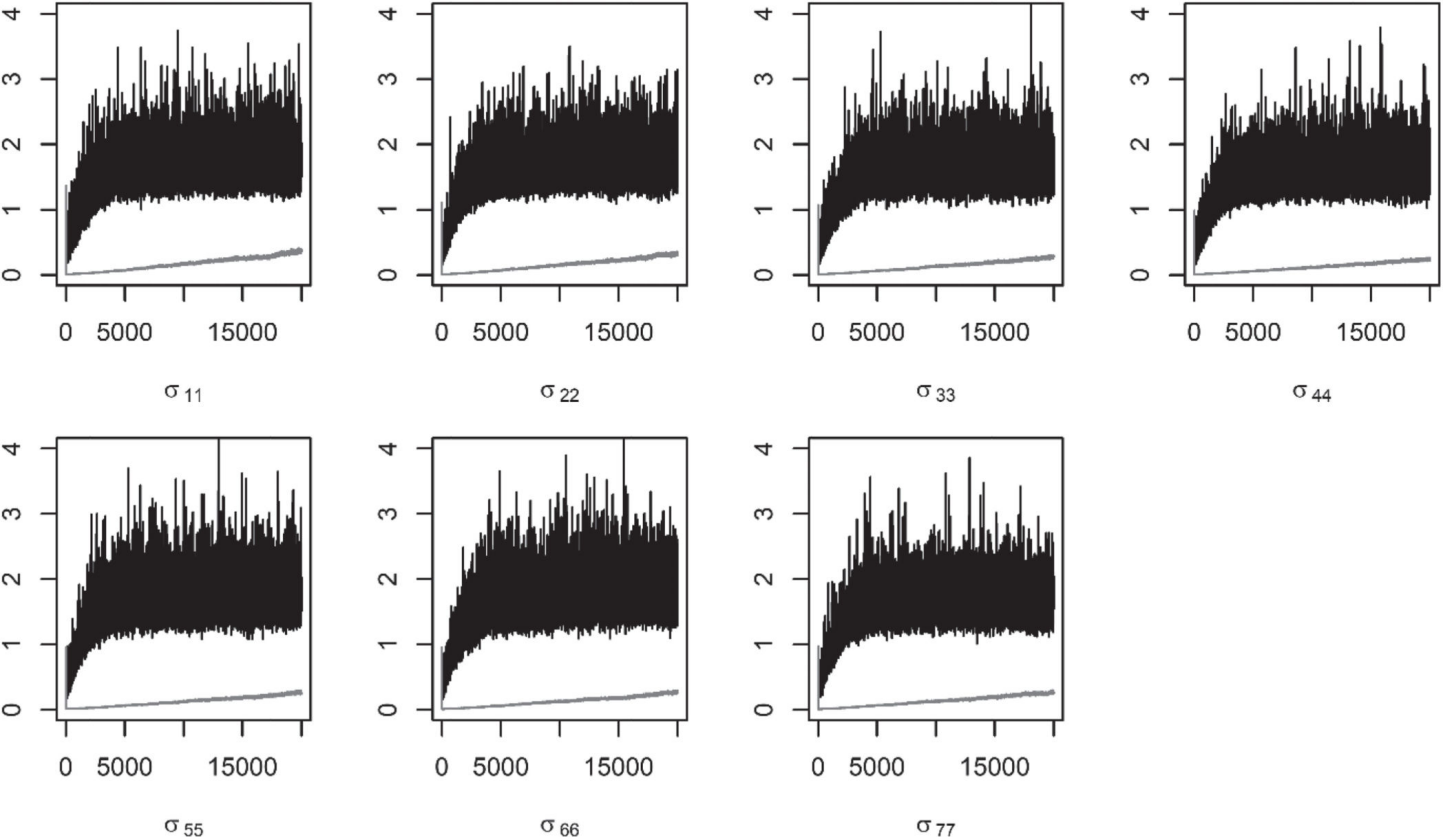
The trace plots for seven redundant variance parameters for the RLMS-HSE data. The black lines denote those from the PX-GSM algorithm while the gray lines denote those from the PX-GS algorithm.

Figure 5 shows the trace plots of the seven redundant variance parameters for the PX-GS and PX-GSM algorithms. As can be observed, those from the PX-GS algorithm do not converge after 20,000 iterations while the PX-GSM algorithm stabilizes after 5,000 iterations. Based on our investigation for the simulation studies with sample size being 2000 in Section 4, the PX-GS algorithm tends to produce biased estimation for the regression parameters, cut-points, and correlations. This explains that the PX-GS algorithm has the smallest estimated values for the repression parameters in Table 3.

**Figure 6.**
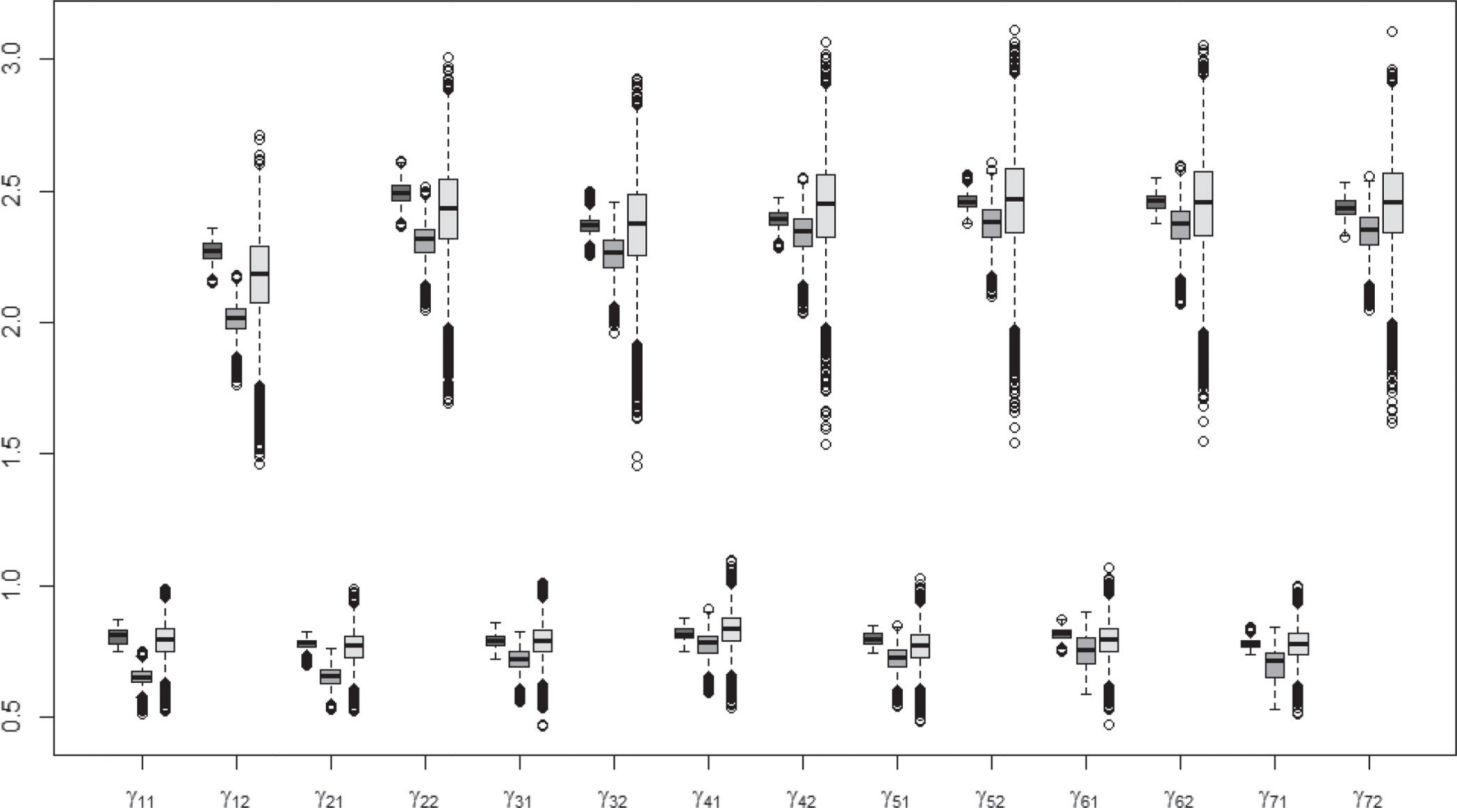
The boxplots for the cut-points based on 15,000 iterations. The dark grey indicates the PX-MH algorithm, the medium grey indicates the PX-GS algorithm, and the light grey indicates the PX-GSM algorithm.

**Figure 7.**
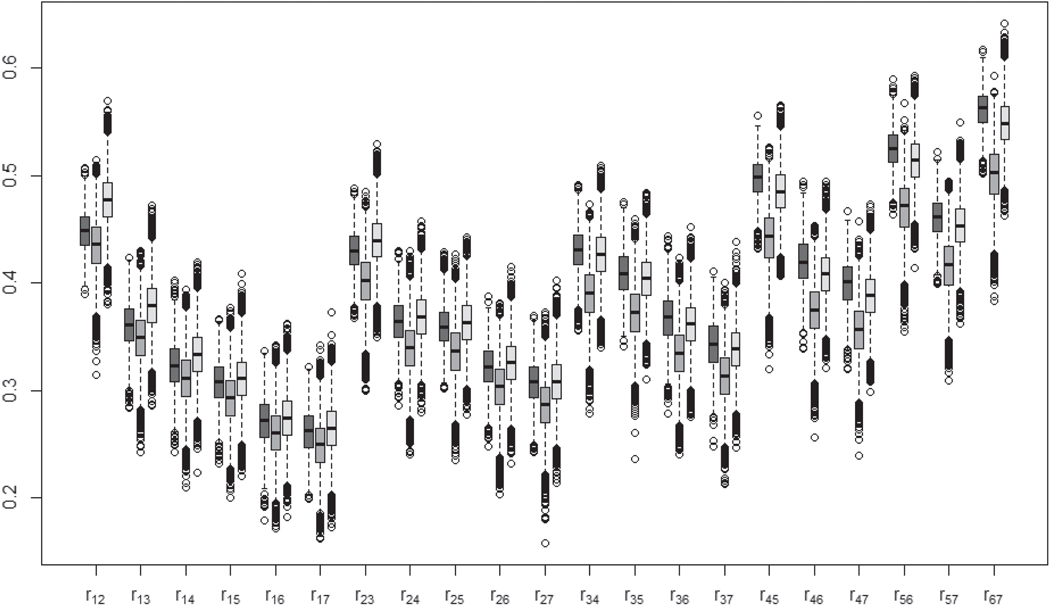
The boxplots for correlations based on 15,000 iterations. The dark grey indicates the PX-MH algorithm, the medium grey indicates the PX-GS algorithm, and the light grey indicates the PX-GSM algorithm.

Figures 6 and 7, the boxplots for the cut-points and correlations, illustrate that the PX-MH and PX-GSM algorithms produce similar estimated cut-points and correlations while the PX-GS algorithm produce the smallest estimated values, suggesting that the slow convergence of the redundant variance parameters brings a significant effect on the estimation of those parameters. The PX-GSM algorithm produces the largest estimated standard deviation for each cut-point (Figure 6), suggesting the PX-GSM has better mixing and faster converge than the other two algorithms (as illustrated in Figure 3 for the simulation studies). The estimated correlations (Figure 7) decrease with time varying from 0.25 to 0.56 with roughly similar estimated standard deviations for all three algorithms. Detailed posterior estimated means and standard deviations for the cut-points and correlations are presented in Supplemental Tables 1 and 2.

## 6. Discussion

In this article, we investigate the PX-MH algorithm based on the identifiable multivariate probit model and the PX-GS and PX-GSM algorithms based on the non-identifiable multivariate probit model.

Both the simulation studies and the application to the RLMS-HSE study show that the PX-GSM algorithm with marginalization of the redundant variance parameters has better mixing and faster convergence for each estimated quantity than the other two algorithms. For data with large sample size, the PX-GS algorithm may produce biased estimation due to the slow convergence of the redundant variance parameters.

The PX-MH algorithm based on the identifiable model produces consistent estimated quantities for both sample sizes being 500 and 2000. However, it converges much slower that the PX-GS and PX-GSM algorithms in general.

The effects of the priors are not discussed in this manuscript since the main focus of this investigation is to compare the convergence property of the identifiable and non-identifiable models. For 5-dimensional data, the prior investigation was conducted by Zhang (2022) and showed that the priors have effects for small to moderate sample sizes, such as 500, but do not have significant effect for large sample sizes, such as 2000. However, there is no investigation regarding the dependence of the results on the priors of both the identified and non-identified parameters for data with high dimensions. This may become one of our future research works.

Our investigation suggests that construction of non-identifiable models should be considered in developing efficient MCMC methods. However, the developed MCMC methods without marginalization of redundant parameters may produce misleading results due to slow convergence of the redundant parameters, especially for data with large sample size. Therefore, marginalization of the redundant parameters should be considered in developing MCMC methods based on the non-identifiable models.

## Supplementary Material

Supplementary

Supplemental data for this article is available online at https://doi.org/10.1080/03610926.2024.2425738

## Figures and Tables

**Table 1.** Averaged posterior means (mean), standard deviations (*SD*), and 95% credible interval coverage probabilities (CP%) with sample size of 500 based on 500 generated datasets.

	True values	PX-MH			PX-GS			PX-GSM		
		Mean	*SD*	CP%	Mean	*SD*	CP%	Mean	*SD*	CP%

$\beta_{1}$	0.5	0.50	0.06	92.8	0.50	0.06	93.2	0.5	0.08	99.6
$\beta_{2}$	0.5	0.51	0.06	93.6	0.50	0.06	95.6	0.51	0.09	99.8
$\beta_{3}$	1.0	0.99	0.06	93.8	1.0	0.07	95.8	1.00	0.10	99
$\beta_{4}$	1.0	1.0	0.07	94.4	0.99	0.07	94.2	1.01	0.11	99.2
$\beta_{5}$	1.5	1.48	0.08	90.0	1.47	0.09	94.6	1.53	0.13	99.2
$\gamma_{11}$	0.5	0.5	0.04	90.6	0.50	0.05	93.6	0.05	0.07	99.8
$\gamma_{12}$	1.0	1.01	0.06	92.4	1.01	0.06	93.4	1.00	0.13	100
$\gamma_{21}$	0.1	0.52	0.05	92.8	0.50	0.05	95.8	0.51	0.08	100
$\gamma_{22}$	1.0	1.03	0.06	90.2	1.01	0.06	94.4	1.02	0.14	100
$\gamma_{31}$	1.0	0.99	0.06	90.4	1.00	0.06	95	1.01	0.10	99.6
$\gamma_{32}$	2.0	2.00	0.08	92.4	2.01	0.09	93.2	2.01	0.18	100
$\gamma_{41}$	1.0	1.00	0.06	93.2	0.99	0.06	94	1.02	0.10	100
$\gamma_{42}$	2.0	1.99	0.08	93.4	1.99	0.09	95.6	2.02	0.18	100
$\gamma_{51}$	1.5	1.47	0.07	85.6	1.46	0.09	93.4	1.53	0.13	99.2
$\gamma_{52}$	2.5	2.48	0.09	88.2	2.46	0.10	93.2	2.53	0.20	99.8
$r_{12}$	0.5	0.49	0.04	92.4	0.49	0.04	92.6	0.49	0.04	93.6
$r_{13}$	0.25	0.24	0.05	94.6	0.24	0.05	95	0.25	0.04	95.4
$r_{14}$	0.125	0.11	0.05	94.6	0.12	0.05	94.6	0.12	0.05	94.4
$r_{15}$	0.0625	0.05	0.05	93.6	0.06	0.05	94	0.06	0.05	93.6
$r_{23}$	0.5	0.48	0.04	95.6	0.49	0.04	96.4	0.49	0.04	96.8
$r_{24}$	0.25	0.24	0.05	94.6	0.24	0.05	95.4	0.24	0.05	95
$r_{25}$	0.125	0.12	0.05	95.6	0.12	0.05	95.8	0.12	0.05	94.2
$r_{34}$	0.5	0.49	0.04	94.6	0.48	0.04	93.2	0.49	0.04	94.2
$r_{35}$	0.25	0.24	0.05	95.4	0.24	0.05	96	0.25	0.05	96.8
$r_{45}$	0.5	0.49	0.04	95	0.47	0.04	94	0.49	0.04	94

**Table 2.** Averaged posterior means (mean), standard deviations (*SD*), and 95% credible interval coverage probabilities (CP%) with sample size 2000 based on 500 generated datasets.

	True values	PX-MH			PX-GS			PX-GSM		
		Mean	*SD*	CP%	Mean	*SD*	CP%	Mean	*SD*	CP%

$\beta_{1}$	0.5	0.50	0.03	95	0.47	0.03	91.6	0.51	0.07	100
$\beta_{2}$	0.5	0.49	0.03	93	0.48	0.03	91.6	0.50	0.07	100
$\beta_{3}$	1.0	1.00	0.03	93.8	0.93	0.05	69.2	0.99	0.09	100
$\beta_{4}$	1.0	1.00	0.03	92	0.92	0.05	54.6	0.99	0.09	100
$\beta_{5}$	1.5	1.48	0.04	88.2	1.31	0.08	6	1.48	0.11	100
$\gamma_{11}$	0.5	0.5	0.02	90.6	0.47	0.03	76.4	0.51	0.07	100
$\gamma_{12}$	1.0	1.0	0.03	87.2	0.95	0.04	81.2	1.01	0.13	100
$\gamma_{21}$	0.1	0.49	0.02	86.2	0.47	0.03	81	0.50	0.06	100
$\gamma_{22}$	1.0	0.99	0.03	85.2	0.96	0.04	97.8	1.01	0.12	100
$\gamma_{31}$	1.0	1.0	0.03	86.2	0.91	0.05	53.4	0.98	0.09	100
$\gamma_{32}$	2.0	2.0	0.04	90.4	1.90	0.06	58.6	1.98	0.17	100
$\gamma_{41}$	1.0	1.0	0.03	89.8	0.90	0.05	30.8	0.99	0.09	100
$\gamma_{42}$	2.0	2.0	0.04	86.8	1.88	0.07	45.2	1.99	0.17	100
$\gamma_{51}$	1.5	1.48	0.04	80.8	1.27	0.09	3	1.47	0.12	100
$\gamma_{52}$	2.5	2.48	0.05	84.6	2.25	0.11	4.6	2.48	0.18	100
$r_{12}$	0.5	0.50	0.02	93.2	0.48	0.02	86.6	0.50	0.03	97.6
$r_{13}$	0.25	0.25	0.02	95	0.24	0.03	94.6	0.25	0.03	97.4
$r_{14}$	0.125	0.12	0.03	96	0.12	0.03	97	0.13	0.03	96.4
$r_{15}$	0.0625	0.06	0.03	95.2	0.06	0.03	96.6	0.06	0.03	95.8
$r_{23}$	0.5	0.50	0.02	93.4	0.47	0.02	77.6	0.50	0.02	97.6
$r_{24}$	0.25	0.25	0.02	96.2	0.24	0.03	93.8	0.25	0.03	98
$r_{25}$	0.125	0.12	0.03	95.6	0.11	0.03	95.4	0.12	0.03	96.4
$r_{34}$	0.5	0.5	0.02	93.4	0.46	0.03	59.6	0.5	0.02	97.6
$r_{35}$	0.25	0.25	0.02	95.6	0.22	0.03	86.2	0.25	0.03	96.6
$r_{45}$	0.5	0.5	0.02	94.2	0.43	0.03	26	0.5	0.02	98.4

**Table 3.** The estimated posterior means with standard deviations and the 95% credible intervals for the regression parameters of the RLMS-HSE data.

	PX-MH	PX-GS	PX-GSM

$\beta_{1}$: gender Mean (*SD*); 95% CI	−0.039 (0.030) (−0.098, 0.018)	−0.034 (0.026) (−0.084, 0.018)	−0.038 (0.030) (−0.098, 0.022)
$\beta_{2}$: education Mean (*SD*); 95% CI	0.089 (0.03) (0.028, 0.149)	0.080 (0.027) (0.028, 0.134)	0.087 (0.031) (0.028, 0.148)
$\beta_{3}$: marital status Mean (*SD*); 95% CI	0.261 (0.03) (0.200, 0.322)	0.226 (0.028) (0.172, 0.280)	0.257 (0.035) (0.191, 0.326)
$\beta_{4}$: age Mean (*SD*); 95% CI	0.005 (0.001) (0.003, 0.008)	0.004 (0.001) (0.002, 0.006)	0.006 (0.001) (0.003 0.009)

## References

[R1] Albert JH, and Chib S 1993. Bayesian analysis of binary and polychotomous response data. Journal of the American Statistical Association 88 (422):669–79. doi: 10.2307/2290350.

[R2] Albert JH, Chib S 2001. Sequential Ordinal Modeling with Applications to Survival Data. Biometrics 57 (3):829–836. doi: 10.1111/j.0006-341X.2001.00829.x. 11550934

[R3] Ashford T, and Sowden RR 1970. Multivariate probit analysis. Biometrics 26 (3):535–46. doi: 10.2307/2529107. 5480663

[R4] Chib S, and Greenberg E 1995. Understanding the Metropolis-Hastings algorithm. The American Statistician 49 (4):327–35. doi: 10.2307/2684568.

[R5] Chib S, and Greenberg E 1998. Analysis of multivariate probit models. Biometrika 85 (2):347–61. doi: 10.1093/biomet/85.2.347.

[R6] Drasgow F 2004. Polychoric and polyserial correlations. In Encyclopedia of Statistical Sciences, edited by Kotz L and Johnson N, 68–74. New York: Wiley.

[R7] Eberly LE, and Carlin BP 2000. Identifiability and convergence issues for Markov chain Monte Carlo fitting of spatial models. Statistics in Medicine 19 (17–18):2279–94. doi: 10.1002/1097-0258(20000915/30)19:17/18<2279::AID-SIM569>3.0.CO;2-R. 10960853

[R8] Edwards Y, and Allenby G 2003. Multivariate analysis of multiple response data. Journal of Marketing Research 40 (3):321–34. doi: 10.1509/jmkr.40.3.321.19233.

[R9] Imai K, and van Dyk DA 2005. A Bayesian analysis of the multinomial probit model using marginal data augmentation. Journal of Econometrics 124 (2):311–34. doi: 10.1016/j.jeconom.2004.02.002.

[R10] Kozyreva P, Kosolapov M, and Popkin BM 2016. Data resource profile: The Russia Longitudinal Monitoring Survey—Higher School of Economics (RLMS-HSE) Phase II: Monitoring the economic and health situation in Russia, 1994–2013. International Journal of Epidemiology 45 (2):395–401. doi: 10.1093/ije/dyv357. 26874929 PMC5007614

[R11] Lawrence E, Liu C, Bingham D, and Nair VN 2008. Bayesian Inference for multivariate ordinal data using parameter expansion. Technometrics 50 (2):182–91. doi: 10.1198/004017008000000064.

[R12] Lewandowski A, Liu C, and Wiel SV 2010. Parameter expansion and efficient inference. Statistical Science 25 (4):533–44. doi: 10.1214/10-STS348.

[R13] Liu C, Rubin DB, and Wu Y 1998. Parameter expansion to accelerate EM: The PX-EM algorithm. Biometrika 85 (4):755–70. doi: 10.1093/biomet/85.4.755.

[R14] Liu J 1994. The collapsed Gibbs sampler in Bayesian computations with applications to a gene regulation problem. Journal of the American Statistical Association 89 (427):958–66. doi: 10.2307/2290921.

[R15] Liu J, and Wu Y 1999. Parameter expansion for data augmentation. Journal of the American Statistical Association 94 (448):1264–74. doi: 10.1080/01621459.1999.10473879.

[R16] Liu X, and Daniels MJ 2006. A new algorithm for simulating a correlation matrix based on parameter expansion and reparameterization. Journal of Computational and Graphical Statistics 15 (4):897–914. doi: 10.1198/106186006X160681.

[R17] MacEachern SN 2007. Comment on article by Jain and Neal. Bayesian Analysis 2 (3):483–94. doi: 10.1214/07-BA219C.

[R18] Meng X-L, and van Dyk DA 1999. Seeking efficient data augmentation schemes via conditional and marginal augmentation. Biometrika 86 (2):301–20. doi: 10.1093/biomet/86.2.301.

[R19] Nobile A 1998. A hybrid Markov chain for the Bayesian analysis of the multinomial probit model. Statistics and Computing 8 (3):229–42. doi: 10.1023/A:1008905311214.

[R20] Pearson K 1900. Mathematical contributions to the theory of evolution. VII. On the correlation of characters not quantitatively measurable. Philosophical Transactions of the Royal Society of London A 195:1–47.

[R21] Plummer M, Best N, Cowles K, and Vines K 2006. CODA: Convergence diagnosis and output analysis for MCMC. R News 6:7–11.

[R22] Shariati MM, Korsgaard IR, and Sorensen D 2009. Identifiability of parameters and behavior of MCMC chains: A case study using the reaction norm model. Journal of Animal Breeding and Genetics 126 (2):92–102. doi: 10.1111/j.1439-0388.2008.00773.x. 19320765

[R23] Smith BJ 2007. boa: An R package for MCMC output convergence assessment and posterior inference. Journal of Statistical Software 21 (11):1–37. doi: 10.18637/jss.v021.i11.

[R24] Van Dyk DA 2010. Marginal Markov chain Monte Carlo methods. Statistica Sinica 20:1423–54.

[R25] Zhang X 2020. Parameter-expanded data augmentation for analyzing correlated binary data using multivariate probit models. Statistics in Medicine 39 (25):3637–52. doi: 10.1002/sim.8685. 32706458

[R26] Zhang X 2022. Bayesian analysis of longitudinal ordinal data using non-identifiable multivariate probit models. Journal of Mathematics and Statistics 18 (1):163–75. doi: 10.3844/jmssp.2022.163.175.

[R27] Zhang X, Boscardin WJ, and Belin T 2006. Sampling correlation matrices in Bayesian models with correlated latent variables. Journal of Computational and Graphical Statistics 15 (4):880–96. doi: 10.1198/106186006X160050.

