# Supplementary material for: Identifiability and convergence behavior for Markov chain Monte Carlo using multivariate probit models
Source: Commun Stat Theory Methods. Author manuscript; Available in PMC 2025 Aug 21. (PMC12366764; doi:10.1080/03610926.2024.2425738)
Supplement: Supplementary [file NIHMS2099078-supplement-Supplementary.pdf]

Supplementary Table 1: The estimated posterior means with standard deviations of the cut-points for the RLMS-HSE data.

Parameters	PX-MH	PX-GS	PX-GSM
γ_{11}	0.81	0.65	0.79
	(0.03)	(0.04)	(0.06)
γ_{12}	2.27	2.01	2.17
	(0.04)	(0.06)	(0.16)
γ_{21}	0.78	0.66	0.77
	(0.02)	(0.04)	(0.06)
γ_{22}	2.49	2.31	2.42
	(0.04)	(0.07)	(0.17)
γ_{31}	0.79	0.72	0.79
	(0.03)	(0.04)	(0.06)
γ_{32}	2.37	2.26	2.36
	(0.04)	(0.07)	(0.17)
γ_{41}	0.82	0.77	0.83
	(0.03)	(0.05)	(0.07)
γ_{42}	2.39	2.34	2.44
	(0.03)	(0.08)	(0.18)
γ_{51}	0.80	0.72	0.77
	(0.02)	(0.05)	(0.06)
γ_{52}	2.46	2.37	2.46
	(0.03)	(0.08)	(0.18)
γ_{61}	0.82	0.75	0.79
	(0.02)	(0.05)	(0.07)
γ_{62}	2.46	2.37	2.45
	(0.04)	(0.08)	(0.18)
γ_{71}	0.78	0.75	0.78
	(0.02)	(0.06)	(0.06)
γ_{72}	2.44	2.37	2.45
	(0.04)	(0.08)	(0.17)

Supplementary Table 2: The estimated posterior means with standard deviations of correlations for the RLMS-HSE data.

Parameters	PX-MH	PX-GS	PX-GSM
r_{12}	0.45	0.43	0.48
	(0.02)	(0.02)	(0.02)
r_{13}	0.36	0.35	0.38
	(0.02)	(0.02)	(0.02)
r_{14}	0.32	0.31	0.33
	(0.02)	(0.02)	(0.02)
r_{15}	0.31	0.29	0.31
	(0.02)	(0.02)	(0.02)
r_{16}	0.27	0.26	0.27
	(0.02)	(0.02)	(0.02)
r_{17}	0.26	0.25	0.26
	(0.02)	(0.02)	(0.02)
r_{23}	0.43	0.40	0.44
	(0.02)	(0.03)	(0.02)
r_{24}	0.36	0.34	0.37
	(0.02)	(0.03)	(0.02)
r_{25}	0.36	0.34	0.36
	(0.02)	(0.03)	(0.02)
r_{26}	0.32	0.30	0.33
	(0.02)	(0.02)	(0.02)
r_{27}	0.31	0.29	0.31
	(0.02)	(0.02)	(0.02)
r_{34}	0.43	0.39	0.43
	(0.02)	(0.03)	(0.02)
r_{35}	0.41	0.37	0.40
	(0.02)	(0.03)	(0.02)
r_{36}	0.37	0.33	0.36
	(0.02)	(0.02)	(0.02)
r_{37}	0.34	0.31	0.34
	(0.02)	(0.02)	(0.02)
r_{45}	0.50	0.44	0.48
	(0.02)	(0.03)	(0.02)
r_{46}	0.42	0.37	0.41
	(0.02)	(0.03)	(0.02)
r_{47}	0.40	0.36	0.39
	(0.02)	(0.03)	(0.02)
r_{56}	0.52	0.47	0.51
	(0.02)	(0.03)	(0.02)
r_{57}	0.46	0.42	0.45
	(0.02)	(0.03)	(0.02)
r_{67}	0.56	0.50	0.55
	(0.02)	(0.03)	(0.02)
